# Characterization of the resting-state brain network topology in the 6-hydroxydopamine rat model of Parkinson’s disease

**DOI:** 10.1371/journal.pone.0172394

**Published:** 2017-03-01

**Authors:** Robert Westphal, Camilla Simmons, Michel B. Mesquita, Tobias C. Wood, Steve C. R. Williams, Anthony C. Vernon, Diana Cash

**Affiliations:** 1 Department of Neuroimaging, Institute of Psychiatry, Psychology & Neuroscience, King’s College London, London, United Kingdom; 2 Department of Basic and Clinical Neuroscience, Institute of Psychiatry, Psychology & Neuroscience, King’s College London, London, United Kingdom; University of Texas at Austin, UNITED STATES

## Abstract

Resting-state functional MRI (rsfMRI) is an imaging technology that has recently gained attention for its ability to detect disruptions in functional brain networks in humans, including in patients with Parkinson’s disease (PD), revealing early and widespread brain network abnormalities. This methodology is now readily applicable to experimental animals offering new possibilities for cross-species translational imaging. In this context, we herein describe the application of rsfMRI to the unilaterally-lesioned 6-hydroxydopamine (6-OHDA) rat, a robust experimental model of the dopamine depletion implicated in PD. Using graph theory to analyse the rsfMRI data, we were able to provide meaningful and translatable measures of integrity, influence and segregation of the underlying functional brain architecture. Specifically, we confirm that rats share a similar functional brain network topology as observed in humans, characterised by small-worldness and modularity. Interestingly, we observed significantly reduced functional connectivity in the 6-OHDA rats, primarily in the ipsilateral (lesioned) hemisphere as evidenced by significantly lower node degree, local efficiency and clustering coefficient in the motor, orbital and sensorimotor cortices. In contrast, we found significantly, and bilaterally, increased thalamic functional connectivity in the lesioned rats. The unilateral deficits in the cortex are consistent with the unilateral nature of this model and further support the validity of the rsfMRI technique in rodents. We thereby provide a methodological framework for the investigation of brain networks in other rodent experimental models of PD, as well as of animal models in general, for cross-comparison with human data.

## Introduction

Resting-state functional MRI (rsfMRI) has matured into a technique capable of mapping functional brain networks and visualizing their organization in health and in disease [1, 2]. Since the discovery of these intrinsic brain networks, numerous computational and analytical methods have been developed to map them. Graph theoretical analysis offers a large set of tools for detecting, analysing, and visualizing functional brain architecture [3]. The implementation of graph theory to the analysis of resting-state data has uncovered evidence that portrays a unique functional organisation of the human brain network, which is characterized by small-worldness and modularity [4–7].

RsfMRI has proved valuable for providing novel insights into putative brain network disruptions in neurological and psychiatric diseases such as schizophrenia [8], Alzheimer’s [9], Huntington’s [10] and Parkinson’s disease (PD). In particular, rsfMRI in non-demented patients with both early and advanced PD has provided compelling evidence of decreased functional connectivity and network efficiency in the areas of motor processing such as the supplementary motor areas and primary motor cortex, the thalamus, the basal ganglia, as well as non-motor areas of the visual, the prefrontal and the primary somatosensory cortex [11–15]. That these findings emerge early in the disease course suggests a potential utility of abnormal resting-state brain networks as a biomarker of disease progression.

In recent years, several groups have successfully back-translated rsfMRI methodology into experimental rodent models [16–18]. This offers an opportunity to apply this methodology to rodent models of PD to evaluate their face validity for the human disease. Whilst rodent models of PD that completely capture the human condition are currently lacking, there are a number of models which recapitulate some important features of the human disease, such as dopamine depletion in the striatum following the loss of nigrostriatal dopaminergic neurons due to neurotoxin exposure. As yet, no such rsfMRI datasets exist for even these basic models. Motivated by these facts, the wealth of resting-state human imaging data and the on-going efforts to develop improved rodent experimental models, the goal of the current study was to capture potential changes in resting-state networks using rsfMRI in the unilateral 6-hydroxydopmaine (6-OHDA) lesion rat as a baseline for extrapolation to newer models as these become available. Importantly, this model is very well characterised at the behavioural, pathological and, as done by our recent MRI study, on a structural level [19]. Although not without limitations, it represents a robust experimental model of dopamine depletion due to destruction of the nigro-striatal pathway applicable to end-stage Parkinsonism [20]. We have therefore deployed graph theoretical analysis to explore functional changes in the 6-OHDA rat at the global and local level of analysis. We hypothesized that, accepting anatomical differences between rodents and humans, we would observe disrupted patterns of functional connectivity within motor, sensorimotor and cognitive brain networks, as reported in PD patients [11–13, 21].

## Methods

### Animals

Animals used in this study are the same cohort as used previously [19]. Therefore, no new animals were generated in this study. Briefly, experimental procedures were carried out in adult male Sprague-Dawley rats (260±25g, Harlan, UK; N = 28) that were group-housed at 21±1°C in a 12 hour light:dark cycle with *ad libitum* access to standard rat chow and drinking water. All experiments were conducted in accordance with the Home Office Animals (Scientific procedures) Act, UK, 1986 and were approved by the King’s College London ethical review committee (Designation no. PCD 70/7085, Procedure 5). Every care was taken to reduce the number of animals used and to minimize suffering.

### Unilateral 6-OHDA lesioning procedure

The lesioning procedure was done as previously described [19]. Briefly, rats were randomly divided into either sham-operated controls (N = 12) or a 6-OHDA-lesioned group (N = 16). Prior to surgery, animals were anesthetized (isoflurane 2.5% in oxygen/air 1:4, 1 l/min, Baxter International Inc., Deerfield, IL, USA) and securely placed in a stereotaxic frame (Kopf Instruments, Tujunga, CA, USA). A hole was drilled in the left frontal bone through which the animals in the 6-OHDA group received 12 μg of 6-OHDA (Sigma-Aldrich, St. Louis, MO, USA) dissolved in 4 μl sterile saline (Sigma-Aldrich) with 0.02% ascorbic acid (Sigma-Aldrich) into the left medial forebrain bundle (MFB) at coordinates (mm) AP -4.4, ML 1 and -7.8 from dura, according to the rat stereotaxic atlas [22]. Injection was performed at a rate of 1 μl/min using a Hamilton syringe (Hamilton, Reno, NV, USA) secured in a motorized syringe pump (Harvard Apparatus, Holliston, MA, USA). The needle was kept in place for an additional four minutes before removal to reduce back flow into the injection tract and facilitate proper diffusion of 6-OHDA. Sham-operated animals underwent the same procedure but received 4 μl sterile saline only. Post-operative care included fluid-replacement (4 ml glucosaline solution i.p.) and mashed high-nutrient food pellets during the first week after surgery.

### Behavioural testing: Apomorphine-induced rotation test

Two weeks after surgery, the success of the nigrostriatal lesioning was confirmed by the apomorphine-induced rotational test [23] as described previously, using an automated rotameter [19, 24]. All rats received a subcutaneous injection of apomorphine (0.1 mg/kg in saline, E-Biomed GmbH, Heidelberg, Germany). Ipsiversive and contraversive full body turns were recorded over a period of 60 minutes. Lesioned rats that displayed at least 100 net contraversive turns, indicative of at least ~80% loss of nigral dopaminergic neurons were included for subsequent MRI acquisition and analysis [25–27]. Lesioned rats that did not display this level of rotational behaviour were excluded from the study at this point. On this basis three 6-OHDA rats were excluded leaving 13 6-OHDA rats for the MR imaging procedure.

### Image acquisition

Resting-state functional MRI data were acquired three weeks post-surgery (i.e. one week after the apomorphine-rotational test) using a 7T MRI scanner (Agilent Technologies, Santa Clara, CA, USA) and a birdcage quadrature RF head coil (40 mm diameter). Animals were anesthetized with isoflurane (5% for induction and 1.5% for maintenance, delivered in medical oxygen/air 1:9, 1 l/min; Baxter International Inc., Deerfield, IL, USA) and secured into a head frame attached to the scanner bed and coil. Animals were placed into the coil and then gently moved into the isocentre of the scanner. All animals were anesthetized for the duration of the MRI acquisition. Physiological monitoring comprising of temperature, respiration rate, pulse rate and blood oxygenation levels was conducted throughout the experiment (SA instruments, Stony Brook, NY, USA). Animal core body temperature was maintained at 37±1°C using a thermostat-controlled air-heating unit (SA instruments). The issue of anaesthesia has previously been discussed in rodent rsfMRI experiments [28, 29]. In this context, we note that isoflurane, which we used here, is a potent systemic vasodilator [30], resulting in changes in the baseline cerebral blood flow independent of task-elicited neuronal activity [31]. Nevertheless, this interfering effect of isoflurane on blood oxygenation level dependent (BOLD) signal is strictly dose-dependent [32]. Moreover, the connectional network architecture of the rat brain at rest is almost entirely preserved at 1% isoflurane [33]. Based on these data, all animals were therefore maintained at 1% isoflurane (delivered as above) during acquisition of resting state data.

For rsfMRI, BOLD sensitive T2*-weighted single-shot echo planar images (EPI) were acquired (20 x 1 mm coronal slices, TE/TR 20/1000ms, matrix 64 x 64, voxel size 0.5 x 0.5 x 1 mm, 900 repetitions [volumes], acquisition time 15 min). Anatomical images for image registration purposes were also acquired using a fast spin echo T2-weighted pulse sequence with the following parameters: 40 x 0.5 mm coronal slices, TEeff 60ms, TR 4000ms, matrix 128 x 256, voxel size 0.25 x 0.125 x 0.5 mm, acquisition time 17 min.

### Data pre-processing

4D rsfMRI images were pre-processed using a combination of statistical parametric mapping software (SPM8, http://www.fil.ion.ucl.ac.uk/spm/, Wellcome Department of Cognitive Neurology, London, UK) and FMRIB Software Library (FSL v5.0; http://www.fmrib.ox.ac.uk, Analysis Group, FMRIB, Oxford, UK) and custom-written scripts in MATLAB (MathWorks Inc., Natick, MA, USA).

Before pre-processing, the voxel size of each image was rescaled by a factor of ten for compatibility with analysis software designed for human scale images. Data pre-processing included slice-timing correction, realignment of all 900 volumes to the first volume and spatial normalization to a rat brain MRI template [34] by the use of the anatomical T2-weighted images. Gaussian smoothing of the normalized volumes was done using a full width half maximum kernel of 10 mm (for the rescaled images, 2x in-plane resolution). The first 50 volumes of the 900 volumes of each subject were removed to exclude possible saturation effects of the transversal magnetization during EPI acquisition. In addition, subjects that showed excessive head motion were also excluded. Head motion was estimated by visually inspecting the six rigid-body realignment parameters (i.e. translations and rotations) obtained during SPM’s realignment processing. The translations were required to be less than 2.5 mm (for the rescaled images; minimum to maximum) and the individual rotations less than 2.5 degrees. In this process, one sham rat showed severe movement artifacts and was excluded.

To remove information in the data that are irrelevant or could potentially confound further analysis (i.e. non-physiological artifacts and physiological noise) as suggested in human rsfMRI [35], linear regressions were applied against the rigid-body realignment parameters and cerebrospinal fluid (CSF) signals. Briefly, the CSF signal was obtained using the CSF prior provided by the MRI template (excluding voxels with a probability value below 0.2 to include only voxels with sufficient CSF proportion) as an region of interest [34] and the SPM toolbox MarsBaR (v.0.4; [36]) to extract the average CSF time series of each rat. Band-pass filtering was then performed (0.01<f<0.1 Hz) to reduce the contribution of low frequency scanner drift and high frequency respiratory nuisance parameters.

Finally, the 850 volumes were temporally masked reducing the time course to a total of 300 volumes (five minutes) by including only volumes with no movement or scanning artifacts [37]. This was accomplished by visually inspecting the realignment parameters of the corresponding volumes first and then either taking the first 300 volumes out of 900 volumes when no artifact was present, or combining together artefact-free volumes when artifacts (e.g. spikes or dips) were found. The resulting 300 volumes thus represent non-continuous time series in some instances. In this work, we assume stationary non-dynamic functional networks across the duration of the acquisition. The technique of using non-continuous resting-state data has been proposed previously and shown to be as useful as data from continuous resting-state data [38].

### Graph formation

**Fig 1:**
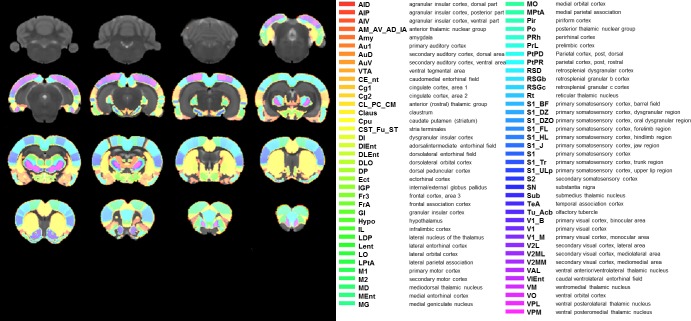
Rat brain parcellation scheme showing 150 ROIs (75 on each hemisphere). Each bilateral ROI is labelled differently and overlaid onto coronal slices of the rat brain template.

In a neurobiological framework, a graph consists of a collection of nodes, here represented by brain regions, and their connecting edges, here represented by the pair-wise functional connections between these brain regions defined by the correlation coefficients. Accordingly, all rat brains were parcellated into 150 regions of interest (ROIs) covering almost the entire brain (75 ROIs for each hemisphere, Fig 1) using a pre-existing template [39], which are defined in Paxinos space [22]. These were then nonlinearly normalized to the rat brain template used for this study [34].

Time course extractions for each ROI were performed using the MarsBaR toolbox (v.0.4; [36]). The pairwise Pearson’s correlation coefficients were calculated across all ROIs generating a 150 x 150 functional connectivity matrix for each rat. From this matrix a binary adjacency graph was formulated by thresholding at a specific sparsity SP (defined as the ratio of edges above a binary threshold relative to the total number of possible edges in the network) [12]. Setting a sparsity-specific threshold normalizes the networks of all subjects to have the same number of edges or wiring cost thereby enabling the examination of the relative network organization in each group [40]. The entire procedure, which resulted in the formation of symmetrical (undirected) and binary graphs, is summarised in S1 Fig.

### Graph measures and analysis

We then derived a series of measures including node degree, clustering coefficient, efficiency, modularity and small-worldness to make inferences about network segregation, integration and influence at the global and local levels of analysis as described previously for undirected and binarized graphs [3, 12]. For the global and local parameter analysis, we chose to threshold each functional connectivity matrix over a wide range of sparsity (0.05 ≤ SP ≤ 0.5) at the interval of 0.05. As such, for brain networks at each SP threshold we calculated small-worldness, global clustering coefficient (normalized to a random reference network), characteristic path length (normalized to a random reference network), global efficiency, number of modules and modularity for a global understanding of the networks, and node degree, clustering coefficient and efficiency at a local level. To compare local network measures independent of a specific threshold we estimated the area under the curve (AUC) for each measure by using a definite integral between two sparsity thresholds (SP = 0.05 and 0.5) as described previously [41, 42].

#### Node degree, clustering coefficient and efficiency

The node degree is a measure of influence and quantifies the number of edges connected to the node [3]. Nodes with a high degree play an important role in the network’s integrity and information flow.

The clustering coefficient characterizes the tendency of a node to be densely interconnected with its topological neighbouring nodes forming segregated functionally specialized clusters [7]. Nodes with a high clustering coefficient are likely to share specialized information with their neighbours. The mean clustering coefficient, often used as a global measure for the level of segregation of the entire network, is defined as the average of local clustering coefficients of all nodes within the network [7].

The nodal efficiency is a measure of integration and characterises how well a node propagates information with the other nodes in a network [43]. The global efficiency on the other hand is often used as a measure of the overall capacity for parallel information transfer and integrated processing [43]. Global efficiency is also the inverse of the path length, which means that global efficiency is greatest when path-length is shortest.

#### Modularity

Typically, highly segregated nodes in the network form specialized community structures or “modules”, which can be detected with sophisticated mathematic algorithms. We applied a greedy optimization algorithm as implemented in GRETNA to identify those modules in both sham lesioned (control) and 6-OHDA rats as previously described [41]. The algorithm statistically detects the optimal community structure of non-overlapping groups of nodes that have maximal numbers of within-module connections and few numbers of between-module links. The modularity Q and the number of modules m were derived from each subject across a range of sparsity (0.05 ≤ SP ≤ 0.5) at the interval of 0.05.

#### Small-worldness

The presence of functionally specialized modules with a high number of intermodularly connected hubs is a network design that is commonly referred to as a small-worldness network [7, 44]. Small-world networks are characterized by the small-world index S, which has often a value much greater than S≫1. The small-world index was calculated according to the following formula as:

$$S=\frac{\overset{-}{C}/{\overset{-}{C}}_{rand}}{L/L_{rand}}$$

where S is the small-world index, $\overset{-}{C}/{\overset{-}{C}}_{rand}$ is the ratio of the mean clustering coefficient of the brain network to the mean clustering coefficient of a random reference network and *L/L_rand_* is the ratio of the characteristic path length (a global measure of integrity) of the same brain network to the characteristic path length of the same random reference network [45].

All global and local graph network measures as well as the generation of random reference networks (the average of 100 random reference networks was used) and node AUC were calculated using the graph theoretical network analysis toolbox GRETNA [42] (v1.2.1 for Windows, https://www.nitrc.org/projects/gretna/).

### Connection-wise comparison analysis

A whole-brain connection-wise comparison analysis was performed using Network Based Statistic Toolbox [46] (NBS; v1.2 for Windows, https://sites.google.com/site/bctnet/comparison/nbs) to identify network edges that were significantly affected by the unilateral 6-OHDA lesion compared to shams. The analysis was done on the unthresholded correlation matrices.

### Statistical analysis

Comparisons of functional connectomes between the 6-OHDA rats and sham rats were performed at the network level by graph-theory analyses and at the individual connection using the network-based statistic. Graphpad Prism (v5.00 for Windows, GraphPad Software, La Jolla, CA, USA, www.graphpad.com) was used to identify group differences in global network properties. Local network differences were identified using GRETNA [42] and NBS [46] was used to determine connections that were significantly affected by nigral dopaminergic neurodegeneration.

**Fig 2:**
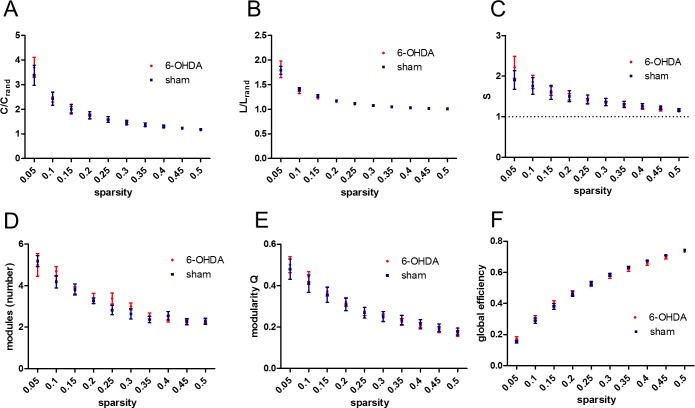
Global network measures of segregation and integration across different network sparsities in 6-OHDA rats and shams. (A) Mean clustering coefficient C normalized to a random reference network. (B) Mean characteristic path length L normalized to a random reference network. (C) Small-worldness S. (D) Number of modules. (E) Modularity Q. (F) Global efficiency. Data are expressed as mean ± SEM. 6-OHDA rats N = 13; sham rats N = 11.

Network-wise comparison on all global network properties was done using a mixed model 2x2 ANOVA with sparsity and group as main effects (Fig 2). Upon confirmation of a significant main effect, post hoc analyses were carried out using Bonferroni’s correction. Significance was assumed at p<0.05. All data are expressed as mean ± SEM.

**Fig 3:**
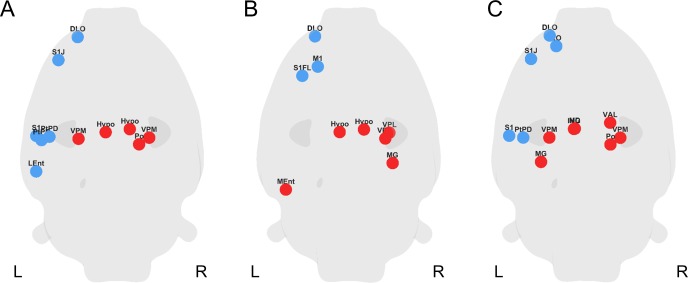
Local differences on a nodal level in 6-OHDA rats compared to shams. Glass brains depicting nodes that showed significantly altered (A) node degree, (B) clustering coefficient and (C) node efficiency in 6-OHDA rats compared to shams. Nodes are represented as spheres and located in the glass brain according to their corresponding anatomical region’s centre mass. Blue spheres depict significantly lower, red spheres significantly higher values in 6-OHDA rats compared to shams. Significance was assessed using two-sample t-test by comparing the AUC of these network measures for each node across all network sparsities (0.05<SP<0.5). A p-value below 0.05 was considered significant (uncorrected). 6-OHDA rats N = 13; sham rats N = 11. Bold L marks the lesion side. R, contralateral side.

Local network-wise comparison was done on the AUC of the nodal network measures (Fig 3). The AUCs for each node for a specific node measure (node degree, clustering coefficient and node efficiency) were compared between groups by using a two-sample t-test. Differences in nodal network measures were considered statistically significant at p<0.05. Multiple comparisons was performed as well using false discovery rate (FDR; q<0.05). Nodes with significant group differences in the local graph measures were depicted as spheres on a glass rat brain positioned in the template space [34] at the centre of mass of the corresponding anatomical brain regions.

**Fig 4:**
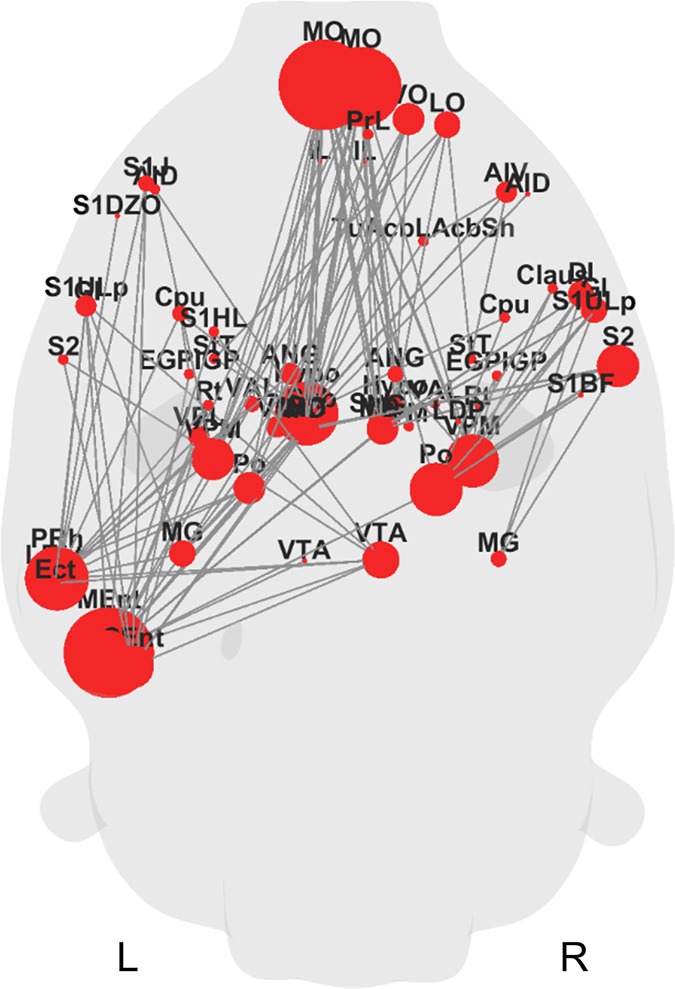
Connection-wise comparison reveals significantly reduced connections in 6-OHDA rats compared shams. Glass brain depicting edges and their associated nodes that showed significantly reduced functional connectivity in 6-OHDA rats in comparison to sham rats. The size of a node represents the number of abnormal connections associated with that node. Significance was assessed using non-parametric permutation tests (N = 10000). A p-value below 0.05 was considered significant (FWER corrected). The results shown were generated at a primary NBS threshold of t = 3.1 with p = 0.0425, FWER corrected. 6-OHDA rats N = 13; sham rats N = 11. Bold L marks the lesion side. R, contralateral side.

Connection-wise comparison was done directly on the raw measure of connectivity using a non-parametric permutation (N = 10000) technique as implemented in NBS to control the family-wise error rate (FWER) when comparing every connection of a network [46] (Fig 4). Edges and their nodes with significant group differences (p<0.05, FWER corrected) in connectivity were depicted on a glass rat brain. The size of a node represents the number of abnormal connections associated with that node.

## Results

### Apomorphine-induced rotations

Thirteen out of sixteen 6-OHDA lesioned rats responded in a robust fashion showing at least 100 contraversive turns (mean: 202.1 turns; SEM: ±9.7) and were therefore deemed to be fully lesioned. Three lesioned rats showed <100 net contraversive turns following apomorphine challenge and were therefore excluded from further experiments and analyses. Therefore, 13 6-OHDA lesioned rats and 12 sham-operated control rats were taken forward to the in vivo rsfMRI acquisition stage.

### Global measures of network topology in 6-OHDA and sham rats

To gain insight into the topological features of both experimental groups and to evaluate the impact of the unilateral 6-OHDA lesioning of the nigrostriatal pathways we first determined global network measures of segregation and integration across a range of sparsity (SP) thresholds such as mean clustering coefficient C (normalized to random reference networks C/C_rand_), mean characteristic path length L (normalized to random reference networks L/L_rand_), small-worldness index S, modularity Q, the number of modules and global efficiency (Fig 2). As expected, we found a significant effect of sparsity on all global network measures analysed (p<0.0001 for all global network measures) but found no significant main effects of either group or group x sparsity interaction in any of these global network measures between 6-OHDA and sham rats. Nonetheless, we confirmed the presence of small-worldness network topology in both groups (as small-world index, S≫1), particularly at a low SP threshold.

### Group differences in node degree, clustering coefficient and efficiency

Having analysed network topology on a global level, we next assessed changes in graph theoretical measures on a local, that is nodal level. To this end, we determined the functional network changes in 6-OHDA rats on the node degree, efficiency and clustering coefficient. Fig 3 shows the glass rat brains depicting the nodes that demonstrated significant differences in these three graph measures in 6-OHDA rats compared to sham rats at p<0.05 (uncorrected) since no results survived FDR correction.

Briefly, at trend level, node degrees were reduced in the left, ipsilateral cortex (e.g. somatosensory cortex and entorhinal cortex) but increased bilaterally in subcortical areas such as hypothalamus or thalamus (Fig 3A) in 6-OHDA rats compared to shams. The clustering coefficients were reduced in the ipsilateral frontal brain areas and increased in subcortical areas (hypothalamus and thalamus) (Fig 3B). Nodal efficiency showed a similar pattern as clustering coefficient and node degree, with a lower efficiency in the ipsilateral cortex (e.g. primary somatosensory cortex and orbital cortex) and higher nodal efficiency in the bilateral thalamus. The results shown in Fig 3 represent the nodes with significant (p<0.05, uncorrected) node degree, clustering coefficient and nodal efficiency values. See S1 Table for a summary of all significant nodes with their corresponding p-values for all three measures.

### Altered functional connectivity in 6-OHDA rats

In order to complement the network-wise graph theoretical analysis, we next performed a whole-brain connection-wise analysis to identify the edges that are significantly altered in 6-OHDA rats compared to shams. The connection-wise comparison analysis revealed significantly reduced connections (NBS primary threshold t = 3.1; p = 0.0425, FWER corrected) in both the ipsilateral and contralateral hemisphere (Fig 4). Interestingly, four out of the five most affected nodes, as indicated by the number of abnormal connections, were located in the ipsilateral hemisphere and include the medial entorhinal cortex (N = 17 abnormal connections), medial orbital cortex (N = 17), hypothalamus (N = 12) and perirhinal cortex (N = 12). The contralateral medial orbital cortex was the only contralateral node in the top five (N = 15). No significantly increased connections in 6-OHDA rats compared to shams were detected.

## Discussion

Here, we applied a graph theoretical analysis to study the topological organization of functional brain networks in the unilaterally lesioned 6-OHDA rat model of PD. We confirm our hypothesis that functional connectivity changes in the 6-OHDA rat resemble those in clinical PD, especially in the ipsilateral cortex, and therefore highlight the utility of in vivo rsfMRI in experimental PD.

### No changes in global network measures in the 6-OHDA rat

We confirm the existence of small-worldness topology in the resting-state networks in isoflurane (1%) anesthetised rats, as has been previously described in both awake and in isoflurane (2%) anesthetised healthy rats [28]. Importantly, small-worldness topology is a fundamental property of the human brain network at rest [6], thus suggesting that the mammalian brain’s global functional organization is evolutionary conserved across these two species. This means that species-specific networks are likely to respond similarly to perturbations and thus validates the use of animal models to study pathological changes in the resting-state networks [47].

We did not however, find any significant main effects of either group or sparsity or group x sparsity interaction in global network measures between the two groups. This may be related to the unilateral nature of the 6-OHDA rat model, where putative compensatory mechanisms in the contralateral hemisphere may cancel out any reductions in functional connectivity in the ipsilateral hemisphere. In other words, a global network analysis may not be able to resolve the regional differences in a unilateral lesion model. In an attempt to further understand the effect of 6-OHDA lesioning in rats on individual brain areas, we performed network analyses on a local level (node and edge level).

### Local functional changes in 6-OHDA rats

In order to obtain a more detailed view of putative functional brain changes in the 6-OHDA lesioned rats we performed local network analyses (node-wise and edge-wise). Overall, the results revealed that the 6-OHDA rats are characterized by a predominantly reduced functional connectivity in the lesioned cortex and an apparently increased functional connectivity in both the ipsilateral and contralateral subcortical areas including the thalamus.

In particular, we found the lesioned hemisphere of 6-OHDA rats to show a significantly reduced node degree, significantly lower clustering coefficient and significantly impaired node efficiency compared to shams, in several areas including the somatosensory and orbital cortices. A low degree along with low clustering coefficient and efficiency is suggestive of reduced integration and influence of ipsilateral nodes indicating that the ipsilateral cortex in particular is increasingly disconnected from the rest of the brain network. This is potentially curious given that the 6-OHDA rat model is based on the prominent *subcortical* dopaminergic cell loss and denervation of the nigro-striatal basal ganglia network. However, our cortical preclinical findings of cortical changes in rodent PD models [48–51] and with our earlier study of 6-OHDA rats where we confirmed cortical dopaminergic denervation by immunohistochemistry, and an associated grey matter loss, particularly in somatosensory and motor cortex, by structural MRI [19]. This is further supported by a fluorodeoxyglucose positron emission tomography (FDG PET) imaging finding of reduced glucose metabolism in the ipsilesional cortex, including the primary motor cortex, in the 6-OHDA rat [27]. Thus, whilst the local level of nodal analysis yielded only uncorrected significant results the fact that structural changes, as discovered by us previously, and functional changes, as observed by others, are evident in the ipsilateral cortex of 6-OHDA rats support the validity of our data. Together, these findings present a picture of a structure-functional relationship connecting the subcortical with cortical dopaminergic denervation, and a resulting reduction in the ipsilesional cortical integration and influence. Thus the changes in functional connectivity may be due to loss of dopaminergic neurons in the substantia nigra and a consequent denervation of other brain areas.

In contrast, we found significantly increased node degree, clustering coefficient and efficiency mostly in the subcortical regions, notably in the bilateral thalamic and hypothalamic areas. Given that these changes are suggested to reflect increased integration and influence of subcortical brain regions, we therefore speculate that our results are related to the emergence of compensatory mechanisms, for example through a plastic remodelling of existing connections into more functionally segregated clusters that could take over particular tasks relating to motor control or coordination, as has been reported in human PD [52]. Indeed, an earlier study showed that the remaining cortex in a rat model of hemispherectomy is still capable of controlling the bilateral motor functions [53]. This suggests that the contralateral cortex could recruit other circuits to maintain bilateral function of the thalamus. Post-mortem studies in rats and cats have shown that efferent fibres from one side of the motor cortex terminate bilaterally in the thalamus [54] therefore supporting this possibility.

The whole brain connection-wise comparison analysis revealed a fuller extent of the functional changes in the 6-OHDA rat. The pattern of the edge map (Fig 4) in 6-OHDA rats suggests a loss of connectivity among many cortical and subcortical brain regions, including a reduced connectivity in the aforementioned ipsilateral cortical areas. The brain area with the highest number of reduced connections is the ipsilateral medial orbital frontal cortex, which, together with findings of reduced node degree, clustering coefficient and efficiency in the ipsilateral dorsolateral orbital cortex, points to an important role of the orbital frontal cortex in the parkinsonian 6-OHDA rat. Function of the orbital cortex in humans remains less well understood, however a regional atrophy of this region has been implicated in clinical PD and linked to the non-motor behavioural symptoms such as anxiety and apathy [55]. Also relevant to our experimental rodent study is the proposed role of the orbital cortex in higher order sensory integration [56] that might link to the findings of sensorimotor deficits in 6-OHDA rats including those of sensorimotor integration such as skilled paw reaching test [57], orientation [58] and corridor tests [59]. Thus, our finding of a reduced ipsilateral cortical functional connectivity in 6-OHDA rats may represent a neuro-functional correlate of these behavioural impairments.

### How do rsfMRI data compare with results from other modalities?

So far only a handful of studies have used graph theory to characterize resting-state functional networks in rodents [28, 60–63] and none has examined the effect of a 6-OHDA lesion. Nevertheless, our data correspond well with previous functional studies in the 6-OHDA rat that used electroencephalography (EEG), ^14^C-2-deoxyglucose autoradiography (2DG), [^18^F] FDG-PET and manganese-enhanced MRI (MEMRI). In particular, consistent with our findings of increased influence and integration of subcortical nodes, increased glucose consumption has been reported in subcortical structures including globus pallidus and SN as measured by 2DG and FDG-PET [64–67]. In contrast, ipsilateral cortical areas such as the somatosensory, auditory and motor cortices showed significantly reduced metabolism [27, 66, 67], which is line with our observation of decreased integration, efficiency and influence of cortical nodes in the lesioned hemisphere.

Previous connectivity studies using EEG have found synchronized beta frequency oscillations in the basal ganglia (BG) including cortical-thalamic, EGP/IGP-thalamic, cortical-striatal, striatal-EGP/IGP and cortical-SN pars reticulata connections [68, 69] indicating increased functional coupling between these brain structures in 6-OHDA rats.

The BG play an important role in motor planning and action selection as well as in reward and addiction behaviour, procedural learning and working memory [70]. Typically, information always flows in a closed loop starting with the cortex, processed in the BG and output back to the frontal cortex through the thalamus. In PD, this pathway is no longer working properly due to an imbalance of dopaminergic transmission in the BG [71, 72]. Our results together with the previous EEG studies point to a special role of the thalamus in the pathology of 6-OHDA rats. Indeed, the thalamus plays an important part in the development of motor symptoms in PD. According to the standard basal ganglia thalamo-cortical circuit model, functional changes in the BG due to the loss of nigral dopaminergic neurons result in an inhibition of the motor thalamus and subsequently reduced excitatory transmission to cortical motor areas [72]. This model is further supported by studies in the 6-OHDA rat that demonstrated reduced spectral EEG power (i.e. increased neuronal desynchronization) in the motor thalamus [73] and increased metabolic activity [74]. Further evidence for altered basal ganglia connectivity has been provided by a previous MEMRI study in 6-OHDA rats reporting decreased axonal transport between EGP and somatosensory cortex but increased SN-anteroventral thalamic-EGP connections [75]. In all, these data suggest that the thalamus receives abnormally high input from other brain areas, potentially inhibitive, leading to a reduced activation of the cortex. Although we were not able to detect significantly increased connections in our edge-wise analysis, our findings of the enhanced subcortical integration and influence of thalamic nodes and the reduced functional connectivity of the ipsilateral cortex support the results converging from other imaging modalities and confirm the classical view of BG connectivity changes in PD.

Together, the high conformity between previous studies using EEG, 2DG/FDG PET and MEMRI and the current rsfMRI study provides further evidence for the utility of resting-state network analysis in PD rat models to examine altered network properties.

### How well does the 6-OHDA rat’s rsfMRI phenotype resembles the human condition?

We set out to analyse the resting state networks in 6-OHDA rats in order to develop a translational tool, allowing better comparison across species, in particular between humans and rodents and vice versa. Ultimately, a thorough phenotyping of animal models of diseases by rsfMRI may help to identify homologous biomarkers that can be used to test effects of pharmacotherapies, or to improve the understanding of cellular basis of functional changes.

Our finding of functional disconnection in the ipsilateral hemisphere in 6-OHDA rats replicates that seen in PD patients. Using graph theory, Gottlich and colleagues found reduced nodal degrees in a visual cortex module along with a decreased centrality in the orbitofrontal cortex in patients with advanced PD [12]. This is in line with a study that found a reduced edge strength in the primary motor area and the extra striate visual cortex in early and moderate staged PD patients [13]. Reduced node strength was also reported in the cingulate, entorhinal and parietal associative cortical brain areas [13, 76] and sensorimotor cortex [15]. Thus, overall reduced connectivity as suggested by the reduced node degree, strength or functional connectivity in human PD is hereby recapitulated in 6-OHDA rats in their lesioned hemisphere lending further support to the use of the 6-OHDA rat as a valid test model of PD.

## Conclusion

We demonstrated the utility of clinically relevant rsfMRI combined with a graph theoretical network analysis to explore functional brain connectivity in rats with a unilateral nigrostriatal neurodegeneration caused by 6-OHDA injection, compared to sham-operated rats. We provide evidence of network connectivity abnormalities in 6-OHDA rats on a local level as well as impaired functional connectivity on the lesioned parkinsonian hemisphere. Interestingly, many of the local functional changes were located in the cortex, which, in this model of nigrostriatal dopaminergic neurodegeneration, highlights the topological knock-on effect of neuronal cell loss in specific subcortical brain areas. Thus, rsfMRI provides an opportunity to explore brain networks in healthy and diseased rodents and thus enables investigation of MRI phenotypes in rodent models to enhance cross-comparison of imaging data in humans and models of PD.

## Supporting information

S1 Fig Flowchart of the graph theoretical network analysis of rsfMRI data. From each subject’s raw images (resting-state functional MRI) the mean BOLD signal time courses of each ROI were extracted. Functional connectivity between any pair of nodes (i.e. edge) was estimated using the Pearson correlation. The resulting correlation coefficients were stored in a correlation matrix for each subject and thresholded at different sparsities to obtain a binary un-weighted symmetrical adjacency matrix. In a neurobiological framework, a graph can be formulated as a set of nodes and edges represented by anatomical brain regions and their functional connectivity, respectively. Once the graph has been formulated, numerous network measures can be derived, such as node degree. Group comparison using non-parametric and parametric statistics can help identify nodes that have significantly different degree in one group compared to another. The result of such group analysis can be best illustrated using a glass brain depicting nodes that are significantly altered.
(TIF)

S1 Table Summary of node level analysis. **Listed are nodes with significantly higher (I) or lower (D) nodal degree clustering coefficient and node efficiency in 6-OHDA rats compared to shams.** Significance was assessed using N = two-sample t-test. Side denotes the hemispheric location of the nodes (L, lesioned hemisphere; R, unlesioned hemisphere). Effect denotes the direction of the effect with D representing decreased and I representing increased effect in relation to the sham group. P represents the p value. A p-value below 0.05 was considered significant (uncorrected). 6-OHDA rats N = 13; sham rats N = 11.
(DOCX)

## Abbreviations

2DG: 14C-2-deoxyglucose autoradiography
6-OHDA: 6-hydroxydopamine
AUC: area under the curve
BG: basal ganglia
BOLD: blood oxygenation level dependent
EEG: electroencephalography
FDG PET: fluorodeoxyglucose positron emission tomography
MEMRI: manganese-enhanced MRI
MFB: medial forebrain bundle
MRI: magnetic resonance imaging
PD: Parkinson's disease
ROI: region(s) of interest
rsfMRI: resting-state functional MRI
SP: sparsity
