# Supplementary figures and images for: Characterization of the resting-state brain network topology in the 6-hydroxydopamine rat model of Parkinson’s disease
Source: PLoS One. 2017 Mar 1;12(3):e0172394. doi: 10.1371/journal.pone.0172394 (PMC5382982; doi:10.1371/journal.pone.0172394)

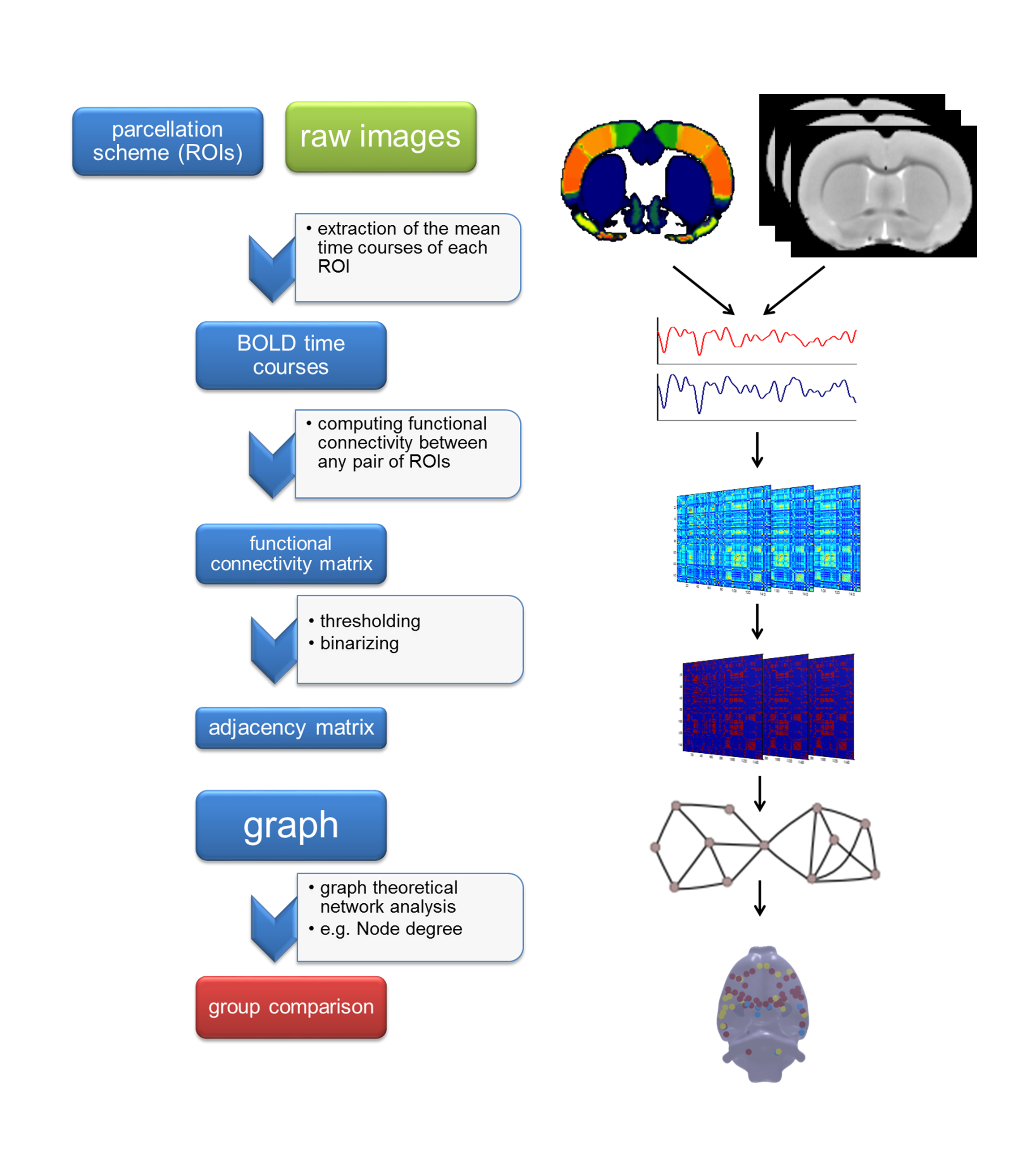

Supplement: S1 Fig — From each subject’s raw images (resting-state functional MRI) the mean BOLD signal time courses of each ROI were extracted. Functional connectivity between any pair of nodes (i.e. edge) was estimated using the Pearson correlation. The resulting correlation coefficients were stored in a correlation matrix for each subject and thresholded at different sparsities to obtain a binary un-weighted symmetrical adjacency matrix. In a neurobiological framework, a graph can be formulated as a set of nodes and edges represented by anatomical brain regions and their functional connectivity, respectively. Once the graph has been formulated, numerous network measures can be derived, such as node degree. Group comparison using non-parametric and parametric statistics can help identify nodes that have significantly different degree in one group compared to another. The result of such group analysis can be best illustrated using a glass brain depicting nodes that are significantly altered. (TIF) [file pone.0172394.s001.tif]
