# Supplementary material for: Characterization of the resting-state brain network topology in the 6-hydroxydopamine rat model of Parkinson’s disease
Source: PLoS One. 2017 Mar 1;12(3):e0172394. doi: 10.1371/journal.pone.0172394 (PMC5382982; doi:10.1371/journal.pone.0172394)
Supplement: S1 Table — Listed are nodes with significantly higher (I) or lower (D) nodal degree clustering coefficient and node efficiency in 6-OHDA rats compared to shams. Significance was assessed using N = two-sample t-test. Side denotes the hemispheric location of the nodes (L, lesioned hemisphere; R, unlesioned hemisphere). Effect denotes the direction of the effect with D representing decreased and I representing increased effect in relation to the sham group. P represents the p value. A p-value below 0.05 was considered significant (uncorrected). 6-OHDA rats N = 13; sham rats N = 11. (DOCX) [file pone.0172394.s002.docx]

|  | node degree | | | clustering coefficient | | | node efficiency | | |
| --- | --- | --- | --- | --- | --- | --- | --- | --- | --- |
| side | node | effect | p | node | effect | p | node | effect | p |
|  |  |  |  |  |  |  |  |  |  |
| L | PtPD | D | 0.005 | MEnt | I | 0.024 | DLO | D | 0.005 |
|  | DLO | D | 0.014 | DLO | D | 0.026 | PtPD | D | 0.018 |
|  | Lent | D | 0.017 | Hypo | I | 0.033 | VPM | I | 0.027 |
|  | S1 | D | 0.021 | M1 | D | 0.041 | MG | I | 0.036 |
|  | PtPR | D | 0.024 | S1FL | D | 0.048 | S1 | D | 0.043 |
|  | Hypo | I | 0.038 |  |  |  | S1J | D | 0.043 |
|  | S1J | D | 0.039 |  |  |  | MD | I | 0.044 |
|  | VPM | I | 0.045 |  |  |  | LO | D | 0.044 |
|  |  |  |  |  |  |  | ING | I | 0.046 |
|  |  |  |  |  |  |  |  |  |  |
| R | Po | I | 0.006 | Hypo | I | 0.003 | Po | I | 0.011 |
|  | VPM | I | 0.007 | VPM | I | 0.033 | VPM | I | 0.016 |
|  | Hypo | I | 0.045 | VPL | I | 0.034 | VAL | I | 0.038 |
|  |  |  |  | MG | I | 0.043 |  |  |  |
